# Temperature-Dependent ^207^Pb Nuclear Magnetic Resonance Spectroscopy: A Spectroscopic Probe for the Local Electronic Structure of Lead Halide Perovskites

**DOI:** 10.1021/acs.chemmater.5c00354

**Published:** 2025-05-02

**Authors:** Sebastian Sabisch, Marcel Aebli, Andrii Kanak, Viktoriia Morad, Simon C. Boehme, Michael Wörle, Leon G. Feld, Christophe Copéret, Maksym V. Kovalenko

**Affiliations:** 1 Department of Chemistry and Applied Biosciences, ETH Zürich, Vladimir-Prelog-Weg 1-5, Zürich CH-8093, Switzerland; 2 Empa-Swiss Federal Laboratories for Materials Science and Technology, Überlandstrasse 129, Dübendorf CH-8600, Switzerland

## Abstract

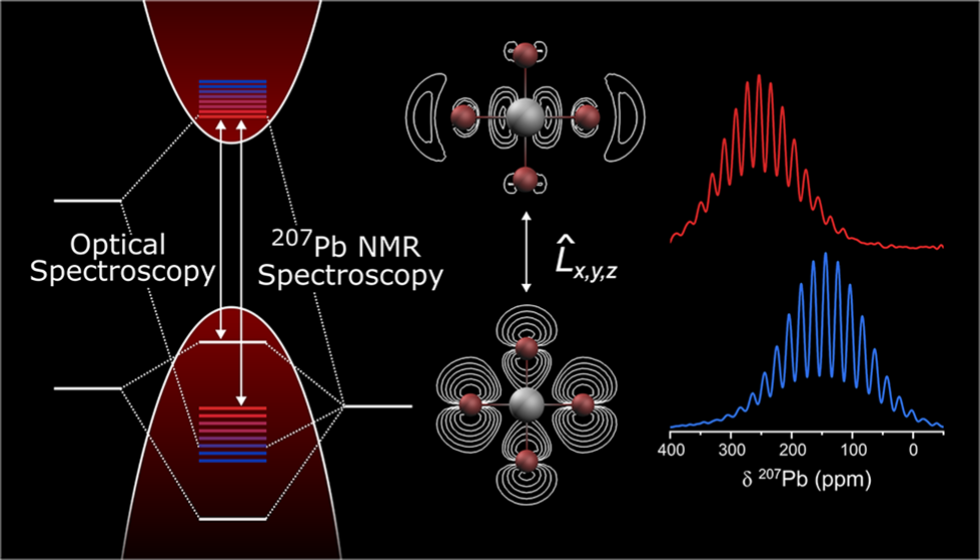

Lead halide perovskites (LHPs) have garnered considerable interest, owing to their advantageous optoelectronic properties and ease of synthesis. However, understanding their intricate structure–property relationships remains challenging, for both bulk and nanoscale forms, such as colloidal quantum dots (QDs). In this study, in addition to conventional characterization by X-ray diffraction and optical absorption, we show that variable temperature solid-state nuclear magnetic resonance spectroscopy, complemented by computational modeling, provides unique insight into the local coordination geometry and electronic structure of LHPs in relation to the moderate change in composition or materials morphology. For CsPbBr_3_ and FAPbBr_3_ in the form of QDs and bulk, we uncover nuanced disparities between their orthorhombic and on-average cubic structures, respectively, reflected in their temperature-dependent ^207^Pb chemical shifts and optical band gaps. Specifically, the mode of thermal expansion, be it the increase of the Pb–Br–Pb angles in the orthorhombic structure or the elongation of the Pb–Br bonds in a cubic lattice, gives rise to an increase of the chemical shift by 0.63 or 1.53 ppm/K and optical band gap by 0.18 or 0.66 meV/K, respectively. Identifying the chemical shift as a spectroscopic descriptor, in particular as a lattice ruler, is highly instrumental also for LHP QDs, capturing the difference between CsPbBr_3_ and FAPbBr_3_. In a broader perspective, establishing relations across spectroscopic and structural descriptors for diverse LHP compositions and morphologies paves the way for informed design strategies in next-generation optoelectronic devices.

## Introduction

Lead halide perovskites (LHPs) of the APbX_3_ family (with A = Cs, methylammonium (MA), formamidinium (FA), among others, and X = Cl, Br, I), consisting of corner-sharing PbX_6_ octahedra with the A-site cation occupying the cuboctahedral void,^1^ have become widely popular because of their notable optoelectronic properties, e.g., large absorption coefficients, tunable band gaps, intrinsic charge transport with long carrier lifetimes,^2−4^ and efficient and narrowband room-temperature emission when produced as quantum dots (QDs).^5−7^ These characteristics, along with facile material synthesis, motivate applications of LHPs in photovoltaics, photo- and hard-radiation detectors, light-emitting diodes, quantum light sources, and lasers.^8−18^ Significant performance improvements in LHP devices are often imparted through seemingly minor structural and compositional adjustments. These include mixing A-site cations or halides and chemical modifications of their surfaces and interfaces.^19−22^ Despite the relatively simple structural space of APbX_3_ compounds, substantial complexity arises from their structural softness and increased structural dynamics, rooted in the low formation energies for both the lattice and some of its defects (such as halide vacancies).^23−25^ The structural softness of LHPs translates, *inter alia*, into increased thermal expansion coefficients and, hence, a steeper temperature dependence of the electronic band gap energy. Furthermore, the optical band gap increases with temperature, unlike the majority of conventional direct-band gap semiconductors.^26,27^ Turning a challenge into a resource, the highly sensitive thermal perturbations to the structural and electronic parameters can, in turn, aid in establishing a structure–property relationship and guide materials design.

Powder and single-crystal X-ray diffraction (XRD) are workhorse techniques for the structural analysis of inorganic solids. However, only limited information is obtained for nanomaterials due to broad and overlapping reflections and other phenomena such as preferential orientation or anisotropic crystal domains. Currently, only the advanced total X-ray scattering techniques can tackle these challenges.^28−31^ In principle, nuclear magnetic resonance (NMR) spectroscopy is a highly potent alternative, naturally set to provide element-specific information about local structure and dynamics in LHPs, regardless of the morphology and long-range order of the sample.^32−34^ Notably, all elements found in LHPs possess NMR-active isotopes (with a nonzero nuclear spin I), namely H, C, N, Cl, Br, I, Cs, and Pb. Some of these nuclei (^14^N, ^35/37^Cl, ^79/81^Br, ^127^I, and ^133^Cs) are quadrupolar (I > 1/2), leading to more complex line shapes and broadened signals due to interaction of the quadrupole moment with the local electric-field gradient.^35,36^ Together with nuclear quadrupolar resonance (NQR) spectroscopy in the case of ^35/37^Cl, ^79/81^Br, ^127^I, solid-state NMR spectroscopy (ssNMR) has successfully enabled the investigation of A-site cation dynamics, binding modes of ligands at the surface of QDs, ligand-induced disorder of the lead halide framework, the nature of Pb-X binding, and structural phase transitions.^20,37−43^^133^Cs NMR of colloids has recently provided first insights into the distribution of local environments in CsPbX_3_ QDs in their native colloidal state.^44^ Finally, ^207^Pb, a spin 1/2 isotope with a natural abundance of 22.1% and a gyromagnetic ratio comparable to ^13^C (0.21 vs 0.25 rel to ^1^H), is a sufficiently receptive NMR isotope (11.8 rel. to ^13^C)^45,46^ for making ^207^Pb NMR of LHPs highly practical. In particular, the ^207^Pb chemical shift has a pronounced temperature sensitivity and has been used as a popular NMR thermometer using Pb(NO_3_)_2_.^47^ Notably, thermal sensitivity was also reported for the chemical shift in LHPs, such as MAPbCl_3_ and CsPbBr_3_.^48,49^ It is noteworthy that the origin of this change of chemical shift remains poorly understood for LHPs at the molecular level,^48−50^ even though it parallels thermally induced changes to the structure and optical property as mentioned above.^26,28^

The chemical shift inherently contains information about the electronic structure, and its temperature dependence parallels the respective evolution of the optical properties and structural parameters.^51,52^ This study rationalizes their interplay on the molecular level using two archetypical LHPs, CsPbBr_3_ and FAPbBr_3_, both previously subject to ssNMR investigations.^53−56^ Due to the different sizes of cations (FA^+^ > Cs^+^) the two compositions represent opposing limits of the stability window. Investigating these materials across various temperatures allows us to accurately fine-tune the physical and electronic structure through thermal expansion rather than compositional changes. We use this thermal perturbation in high-quality bulk materials to correlate distinct temperature-induced changes to crystal structure, such as increased bond lengths in cubic structures or tilting between neighboring octahedra in orthorhombic structures to the respective optical and NMR signatures. Here, we find that the influence of the thermal expansion in FAPbBr_3_ on the band gap and chemical shift is more pronounced than the changes observed for CsPbBr_3_. Going beyond the empirical correlation, we uncover that these properties are rooted in the local electronic structure and can be rationalized by a natural chemical shift analysis and corroborated by investigating the respective band structures. These results point to the antibonding conduction band (CB) and its bonding counterpart rather than the optical band gap as the origin. Using the ability of NMR to probe the local (electronic) structure, we explore the signatures of the corresponding QDs, for which XRD does not readily provide a full structural description. Based on our understanding of the local structure determined by single-crystal XRD on bulk materials and our spectroscopic investigation across a wide range of temperatures, we establish a direct link between the local structure and spectroscopic descriptors.

## Results and Discussion

**Figure 1:**
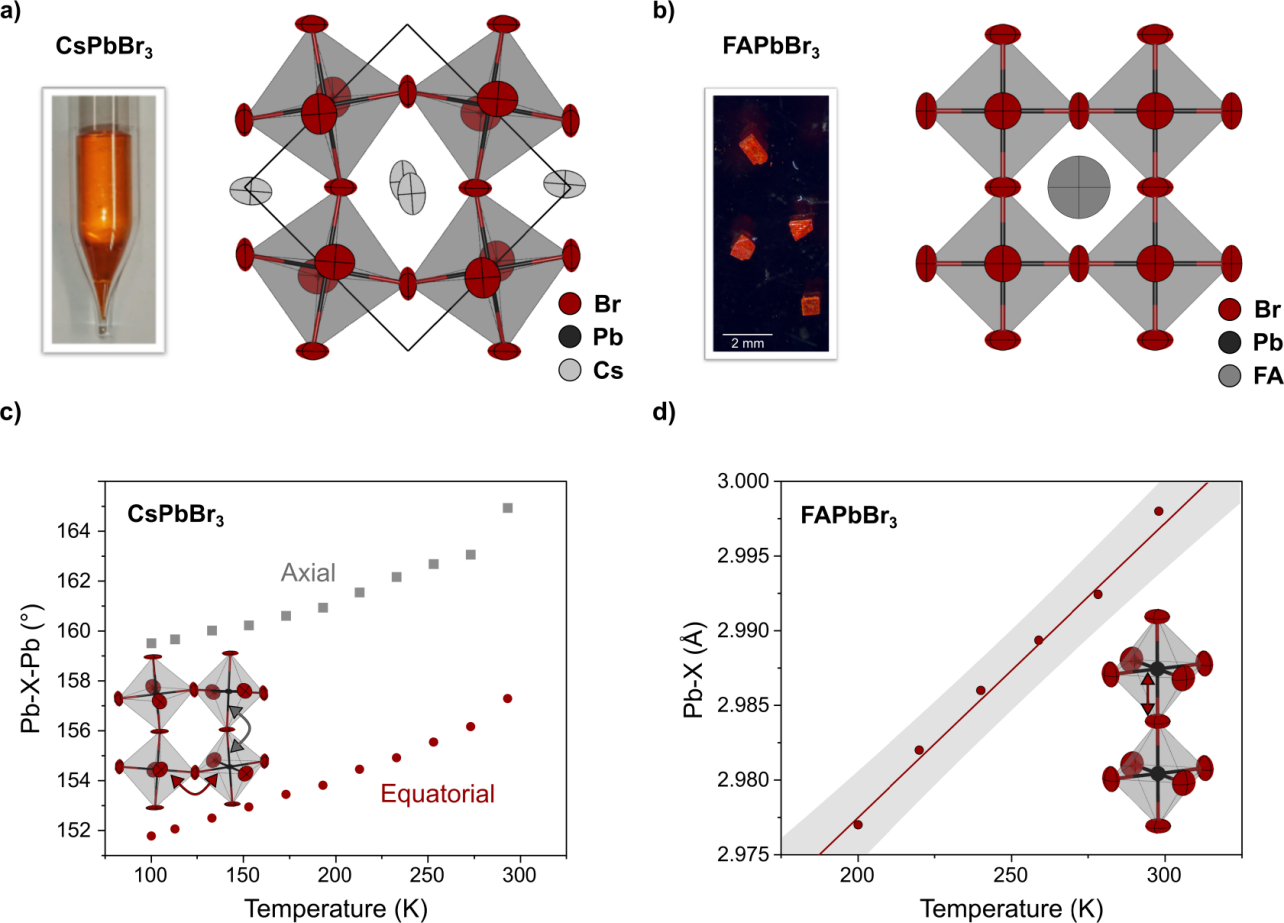
(a) Image and crystal structure of the centimeter-sized melt-grown CsPbBr_3_ ingot used for this study. (b) Image and structure of solution-grown FAPbBr_3_ single crystals. (c) Tilt angles between neighboring PbBr_6_ octahedra along the crystallographic *c* and *a*/*b* directions of CsPbBr_3_, as obtained from single-crystal XRD. (d) Pb–Br distances in FAPbBr_3_ as obtained from single-crystal XRD.

First, the effect of temperature on both the local geometry and the electronic structure was investigated for bulk CsPbBr_3_ and FAPbBr_3_ perovskites. Different A-site cations manifest themselves in different lattice symmetries, optical band gaps, and temperature sensitivities of their optical and structural parameters.^57−59^ Therefore, high-quality samples were prepared by the Bridgman–Stockbarger method for CsPbBr_3,_^60,61^ and the inverse temperature crystallization method for FAPbBr_3_ (Figure 1a,b).^10^ XRD analysis confirmed that CsPbBr_3_ crystallizes in an orthorhombic lattice at room temperature (Figure 1a), *i.e*., all angles between the corner-sharing octahedra deviate from 180°. In contrast, FAPbBr_3_ is cubic at room temperature, i.e., all angles between octahedra being 180°, due to the increase in space occupied by the fast-rotating FA^+^ cation.^62,63^

Single-crystal X-ray diffraction experiments were performed over a temperature range between 100 K and room temperature to decipher the structural origin of the observed changes in the electronic structure. The structure of CsPbBr_3_ is described in the orthorhombic space group *Pnma* between 100 K and room temperature. Twinning is commonly observed as the sample transitioned from cubic to orthorhombic phases during the crystal growth from the melt. Between 200 K and RT, FAPbBr_3_ can be described using a cubic space group *Pm*-3*m* containing a disordered FA^+^ cation. While the cubic FAPbBr_3_ structure shows an isotropic contraction upon cooling (Figure S1), the orthorhombic CsPbBr_3_ structure contracts along the crystallographic *b* and *c* directions but expands along *a* (Figure S2). Due to the slight difference between the a and b axes in the orthorhombic structure, single-crystal diffraction is preferred over powder diffraction to avoid the overlap of reflections. The tilting between neighboring octahedra is extracted from the results of the structure analysis (Figure 1c), revealing that the thermal expansion in CsPbBr_3_ is mediated by a decrease in the octahedral tilting from 152° to 157° along both the crystallographic *a* and *b* direction and from 159 to 165° along the *c* direction, without changes in the average Pb–Br bond length (Figure S3). To the contrary, FAPbBr_3_ expands through elongation of the average lead-halide bond by 2.5 pm between 200 K and RT (Figure 1d) while maintaining a cubic structure and hence no change in the octahedral tilting.

**Figure 2:**
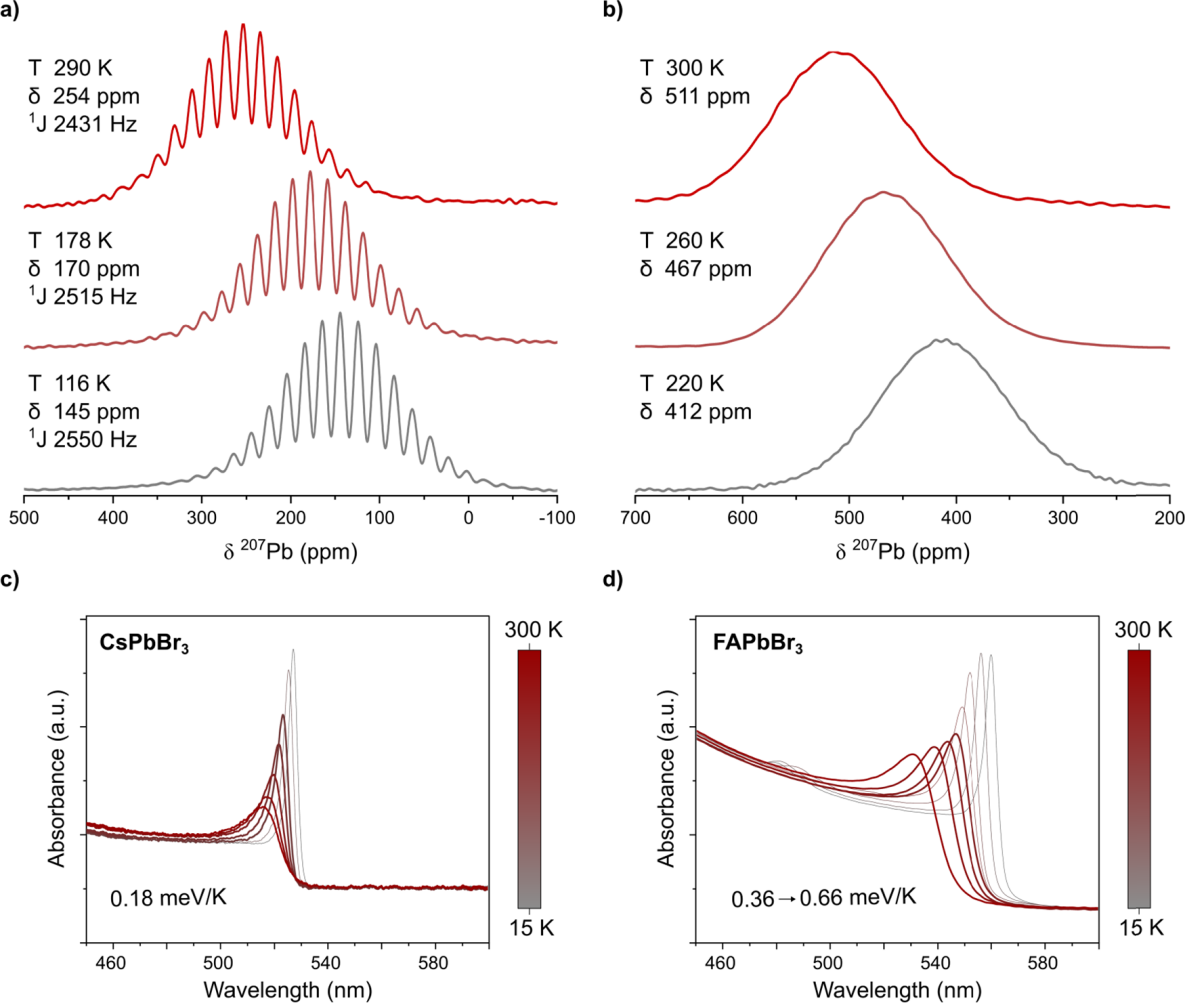
(a, b) ^207^Pb MAS NMR spectra of bulk (a) CsPbBr_3_ and (b) FAPbBr_3_ at various temperatures. (c, d) Temperature-dependent absorption spectra of CsPbBr_3_ and FAPbBr_3_ microcrystalline films on quartz substrates between 15 K (gray) and room temperature (red). Traces corresponding to the temperature range of the respective ssNMR measurements (100–300 and 200–300 K) are highlighted with bold lines.

Next, ^207^Pb ssNMR spectra of both materials were acquired at a field of 14.1 T under magic angle spinning (8 kHz). For both materials, room-temperature spectra exhibit a single line with a chemical shift (δ_iso_) and a full width at half-maximum (fwhm) of δ_iso_ = 253.7(1) ppm/21.5 kHz and δ_iso_ = 510.9(1) ppm/18 kHz for CsPbBr_3_ and FAPbBr_3_, respectively. The observed line broadening and fine structure arise from the J-coupling of lead to the six surrounding bromides (*I* = 3/2), resulting in a multiplicity of 19 (*m* = (2*n* × *I*) + 1).^49^ The difference in the fwhm between the NMR lines of both structures is explained by a smaller J-coupling constant found in FAPbBr_3_ (2335(2) Hz), resolved here for the first time (Figure S4), compared to CsPbBr_3_ (2430.6(4) Hz) (Figure 2a). After the initial assessment of the spectra obtained at room temperature and to leverage the high temperature sensitivity of the Pb chemical shift, ssNMR spectra were collected at temperatures between 100 K and room temperature. The chemical shift of CsPbBr_3_ linearly increases from 100 to 300 K with a slope of 0.630(2) ppm/K. The chemical shift of FAPbBr_3_ increases with a slope of 1.53(3) ppm/K but can only be observed above 220 K due to a first-order phase transition (Figure 2a,b and Figure S5). Below 220 K, the fast relaxation times do not permit an observation of FAPbBr_3_.^49^ Compared to established NMR thermometers, e.g., Pb(NO_3_)_2_, which was used to reference the temperature in all experiments, the temperature dependence of the chemical shift of FAPbBr_3_ is significantly larger, making it the most sensitive lead compound to date (0.7 ppm/K reported for Pb(NO_3_)_2_ and the 0.9 ppm/K reported for MAPbCl_3_).^47,48^

Additionally, it is noteworthy that the J-coupling constant, extracted for CsPbBr_3_, also displays a temperature dependence of −0.561(2) Hz/K, suggesting a change to the local geometry of the PbBr_6_ octahedra. Similar temperature sensitivity was observed for several other compositions, e.g., MAPbCl_3_, MAPbBr_3_, and MAPbI_3_ (Figure S5).

To further investigate the temperature dependence of the electronic structure, we turned to variable-temperature optical absorption spectroscopy. Due to their significant absorption coefficients, thin films (∼80 nm) of both compounds were fabricated by either thermal evaporation (CsPbBr_3_) or spin coating (FAPbBr_3_) on quartz substrates. At 15 K, the absorption spectrum of both compounds comprises a pronounced excitonic feature at the optical band gap (2.40 eV for CsPbBr_3_ and 2.33 eV for FAPbBr_3_), followed by a continuous increase of the absorption toward smaller wavelengths (Figure 2c,d). The optical band gap observed for these thin films is slightly higher compared to the corresponding bulk materials, likely attributable to the microcrystalline morphology present in such films. Both compounds exhibit a blue shift of the absorption edge upon heating to room temperature, consistent with previous reports,^26^ and a broadening of the excitonic feature (Figure 2c,d). The anomalous increase of the band gap with temperature, which opposes the trends observed for conventional semiconductors,^27^ arises from the antibonding nature of both the conduction and valence bands in LHPs, a defining feature that lies at the core of their previously reported defect tolerance.^23,64^

Notably, the slope of the band gap upon heating is more significant for FAPbBr_3_ than CsPbBr_3_. This is in line with the temperature sensitivity of the chemical shift, providing a first indication that the origins of both could be closely related. While the energy of the peak of the excitonic feature and the band gap increase continuously in all-inorganic perovskite with a slope of 0.1816(4) meV/K, the slope observed in the more symmetric hybrid perovskite is larger and more complex. Between 15 and 150 K, a slope of 0.358(1) meV/K is observed, followed by a discontinuity; above 170 K, the slope increases to 0.6612(6) meV/K (Figure 2d and S6). The discontinuity appears at the same point as a first-order phase transition. The difference between the slopes before and after the phase transition (vide infra) provides another strong indication that the temperature dependence of the electronic structure is closely related to the average symmetry of the system, with less symmetric systems being less temperature sensitive.

Measuring the local structure of QDs is crucial because their optical properties depend on both electronic (quantum-size effects) and structural factors. QDs naturally lack long-range order, leading to broad and overlapping reflections in diffraction experiments, limiting their use for a detailed structural analysis. On the other hand, the local probing via ssNMR spectroscopy is much less affected by the lack of periodicity and is sufficiently sensitive in resolving the underlying topological disorders.^32,65^

**Figure 3:**
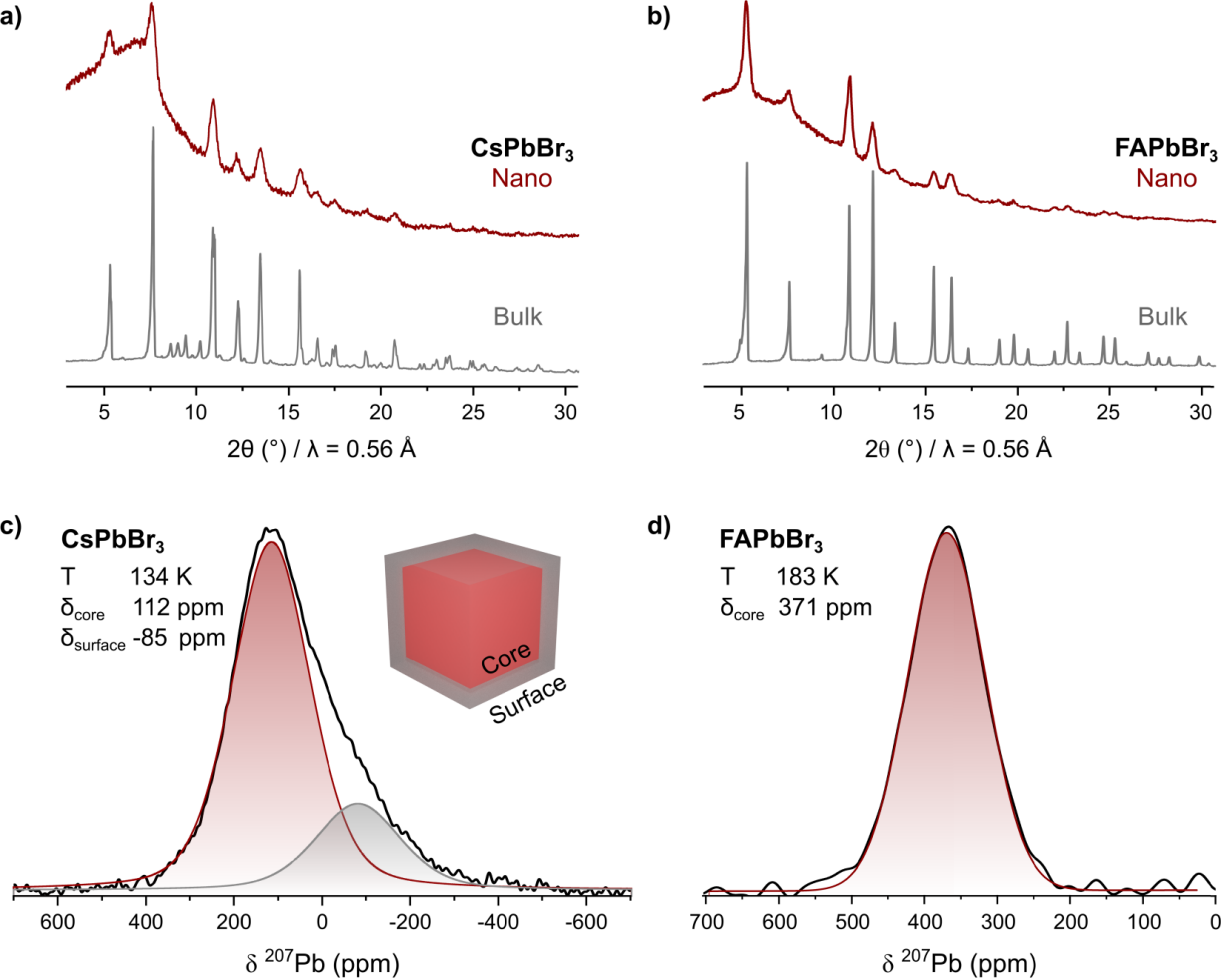
(a, b) Powder XRD patterns of CsPbBr_3_ and FAPbBr_3_ both in their microcrystalline bulk and QD forms, acquired for 56 h with four parallel detectors using a AgK_α1_ (λ = 0.55941 Å) source. (c, d) ^207^Pb MAS NMR spectrum of cuboidal CsPbBr_3_ and FAPbBr_3_ QDs with an edge length of 9 nm at 134 and 183 K, respectively.

The similarity between the structures of bulk and QDs can be deduced from their powder X-ray diffraction patterns. The diffraction patterns of bulk and nanocrystalline CsPbBr_3_ and FAPbBr_3_ were obtained on a benchtop diffractometer equipped with four parallel detectors with an integration time of 56 h (Figure 3a). While the positions of the reflections between both samples are similar, both patterns significantly differ in terms of their broadening and background; the bulk samples show sharp Bragg reflections, while the QDs show broadened and more diffuse reflections combined with a prominent background caused by the solvent (hexane) and ligands. The increased broadening of the reflections in QDs is the result of the small crystalline domains as described by the Scherrer equation.^66^ Additionally, topological disorder causes many features to no longer be detectable, while other reflections start to overlap with neighboring ones, increasing the complexity of the diffractogram even further. This limited resolution in persists even when using synchrotron sources.^29^ Similar phenomena can be observed for FAPbBr_3_; while there are distinct differences between the diffractograms of the cubic and noncubic QDs, the limited information density would not allow an ab initio structure solution nor an accurate determination of the lattice constants (Figure 3a,b).

To compare the local structures of colloidal QDs and bulk crystals, the ^207^Pb NMR spectra of CsPbBr_3_ and FAPbBr_3_ QDs were acquired across the same temperature ranges as their bulk counterparts. The NMR line of the cuboidal CsPbBr_3_ QDs with an edge length of 9 nm appeared at a similar chemical shift as the bulk and showed a slightly broadened peak (fwhm of 25 kHz) with a shoulder upfield (toward lower chemical shifts) shifted by 130 ppm (Figure 3c). Such a shoulder, recently discussed in the context of ^133^Cs NMR,^44^ may be attributed to the ligand-induced disorder on the QD surface. The spectrum of FAPbBr_3_ QDs displays a signal with a slightly higher chemical shift and similar width as the bulk (Figure 3d), but without a shoulder peak. We speculate that the difference arises from a better geometrical fit of the cationic moiety of the ligand headgroup to the surface A-site of FAPbBr_3_, thus causing a lower level of structural distortion upon binding. The striking similarity between the spectra of the QD and bulk samples highlights the similarity of the local environments and the suitability of NMR spectroscopy to investigate nanoscale materials.

**Figure 4:**
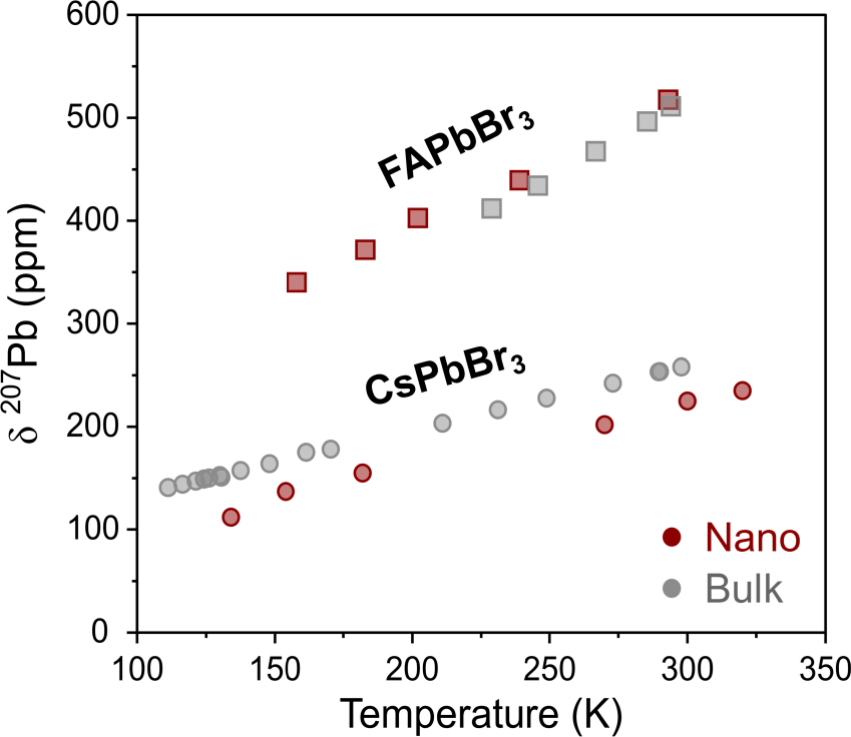
Comparison of the fitted isotropic chemical shifts of bulk and colloidal LHPs at temperatures between 100 K and room temperature.

To elucidate changes to the local structure, we therefore chose to compare the temperature-dependent chemical shifts of the bulk and QD samples of both CsPbBr_3_ and FAPbBr_3_. The temperature-dependent chemical shifts reveal similar slopes between the bulk and QD samples of the same composition with the temperature sensitivity of the cubic structure exceeding the orthorhombic one (Figure 4). These slopes, as elucidated for the bulk materials, are characteristic of the symmetry reflected in the local structure, indicating that the local structure seems to be independent of the sample morphology. These results also allow us to categorize their structure in terms of their temperature-dependent local structure rather than long-range symmetry.

As recently reported, size-dependent effects on the structure can occur for small crystal domain sizes and, therefore, naturally for nanomaterials.^28^ An instance of such a discrepancy is the size-dependent phase transition temperature in FAPbBr_3_, which manifests in a change in the relaxation times, leading to the disappearance of the observable signal. This phase transition temperature for the bulk was observed at around 220 K, consistent with previous reports.^26,49^ The decrease of this phase transition temperature to around 160 K for the QD sample demonstrates that variable-temperature ssNMR can probe size-dependent phase transitions even in colloidal QDs.^26^

To understand the remarkable correlation between the temperature-dependent spectroscopic signatures and structural changes, while considering that the chemical shift intrinsically contains detailed information about the electronic structure of the observed element, here Pb, we analyze the ^207^Pb chemical shift by computational modeling, using, in particular, natural chemical shift analysis.^51,52,67^ Several models based on finite and periodic systems were built directly from the experimentally determined single-crystal structures, validated, and then used to calculate the chemical shielding (σ_Sample_ ≈ σ_Reference_ – δ_Sample_) and its components, i.e., the diamagnetic and paramagnetic shielding terms, which can aid in elucidating the origin of the experimentally observed chemical shift and establishing a detailed understanding of electronic structures.^50,67^ Diamagnetic shielding (σ_dia_) is mostly driven by an isotropic distribution of (mostly core) electrons; it remains mostly constant across a range of structures and leads to shielding (lower chemical shift values). In contrast, in molecular systems, paramagnetic shielding (σ_para_) is mostly related to frontier molecular orbitals and arises from the coupling of occupied and unoccupied orbitals, close in energy and of appropriate symmetry. Most specifically, the paramagnetic shielding, as described by the Ramsey equation (eq 1), scales inversely with the difference in energy between the set of coupled filled and vacant orbitals (σ_para_ ∝ ΔE^–1^).^51,^^67,68^ In addition, this term depends on specific conditions as determined by the symmetry of the angular momentum operator *L̂*, connecting occupied and unoccupied orbitals orthogonal to each other in the presence of a magnetic field.
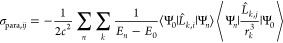
1

In highly symmetric and dynamic coordination environments as present in both CsPbBr_3_ and FAPbBr_3_, specific principal components of the chemical shift tensor (δ_11_ ≥ δ_22_ ≥ δ_33_) and associated chemical shift anisotropy, (Ω = δ_11_ – δ_33_), are not expected to play a significant role due to the local octahedral symmetry around Pb (Ω → 0). Note that some deviations are expected for nuclei at surface layers due to loss of symmetry and as they make up a significant fraction of atoms in a colloidal QD (*ca*. 20% for 8 nm QD size).

With this reasoning, we set out to construct representative models directly derived from the recorded X-ray structures. The geometry of the experimental structures was optimized using periodic DFT, while the lattice parameters were frozen to preserve the temperature of the crystal structure. To avoid an orientation dependence of the FA^+^ cation, it is replaced with Cs^+^, resulting only in a minimal shift of the band energies (Δ*E*_BG_ < 0.1 eV). As the analysis of the chemical shift is based on molecular orbitals, these optimized structures are then used to additionally create two finite cluster models containing either a single octahedron (PbBr_6_) or 27 corner-sharing octahedra (Cs_56_Pb_27_Br_108_). The small cluster models allow for the use of a higher level of theory, including relativistic effects at the spin–orbit level of theory (PBE0, TZ2P, ZORA-SO). The larger cluster is needed to capture the angles between neighboring octahedra, which is crucial for describing thermal expansion in CsPbBr_3_.

**Figure 5:**
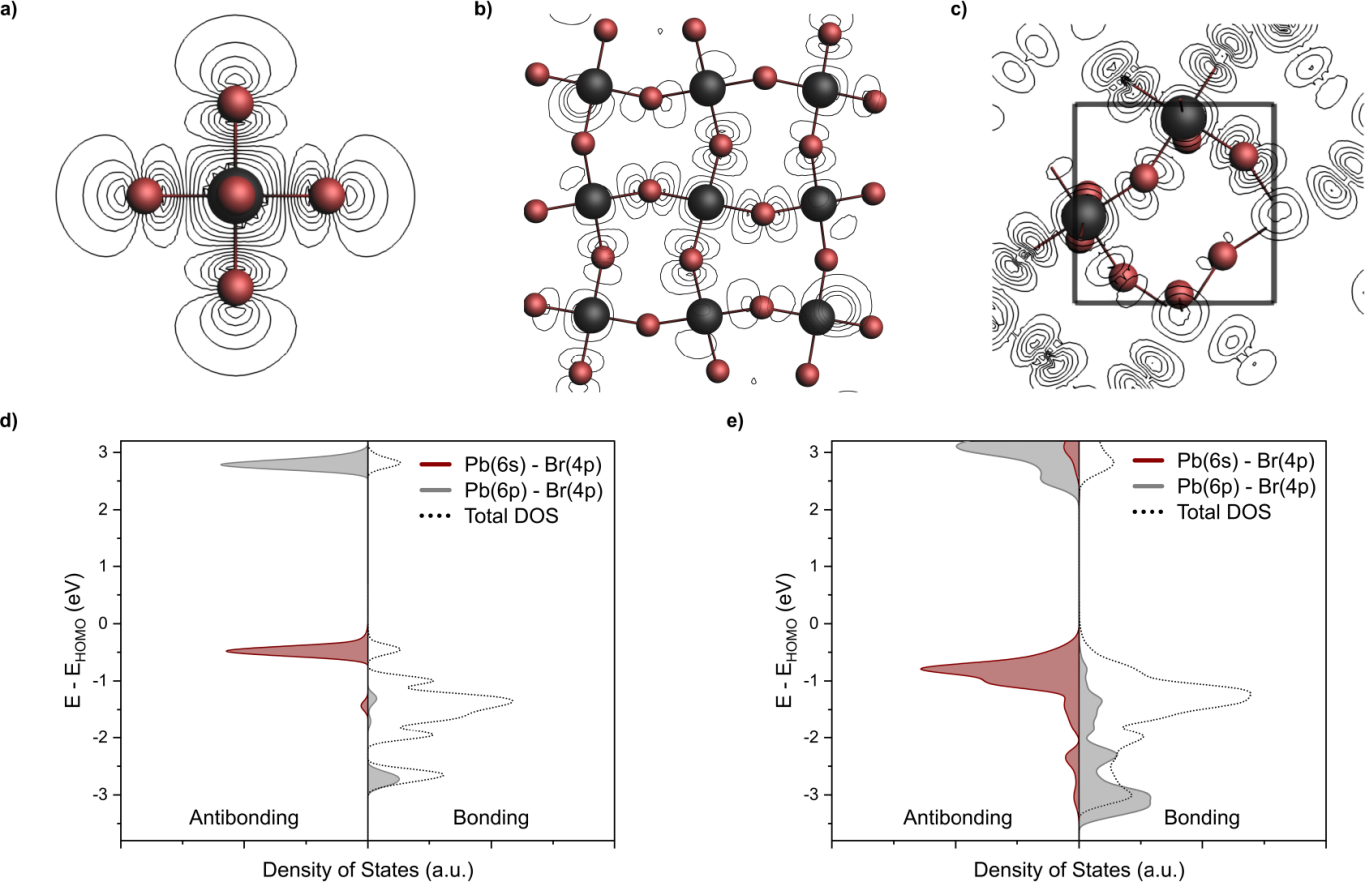
(a–c) Contour representations of the HOMO/VB wave functions of the PbBr_6_, Cs_56_Pb_27_Br_108_ and periodic models consisting of an s-orbital on Pb and p-orbitals on Br. (d, e) Density of states and COOP analysis obtained from the small cluster and periodic model calculated using periodic boundary conditions, showing the qualitative similarity between the models and reduced quantization of the electronic structure with increasing model size.

To confirm the validity of the models and to ensure that the results of the analysis are transferable between finite and periodic models, we first compare the nature of the highest occupied molecular orbital (HOMO) of the cluster models with those of the valence band (VB) edge of the periodic model: both the HOMO of the cluster models and the VB edge of the periodic model are derived from 6s orbitals of Pb and 4p orbitals of Br with an antibonding overlap (Figure 5a–c), while the conduction band (CB) edge and the respective lowest unoccupied molecular orbital (LUMO), which are antibonding as well, are mainly derived from 6p orbitals of Pb and 4p orbitals of Br (Figure 5d,e). While all models show a qualitatively similar density of states, the gap between the HOMO and LUMO (VB to CB) decreases with the size of the model. Additionally, the quantized (separated) nature of the states found for the smallest cluster model already disappears for the larger cluster model (Figure 5d,e). Note that a limitation of these computational models is the absence of local dynamics, which have been known to lower the local symmetry compared to the average structure and therefore also alter the electronic structure. This error could be reduced by either considering a time-averaged band gap or a larger unit cell with local distortions.^30,69^ While fast dynamics are relevant for the relaxation of nuclear spins, the chemical shift represents the time-averaged structure. After establishing the computational models’ validity, we continue analyzing the electronic structure at room temperature to establish a link to the chemical shift.

**Figure 6:**
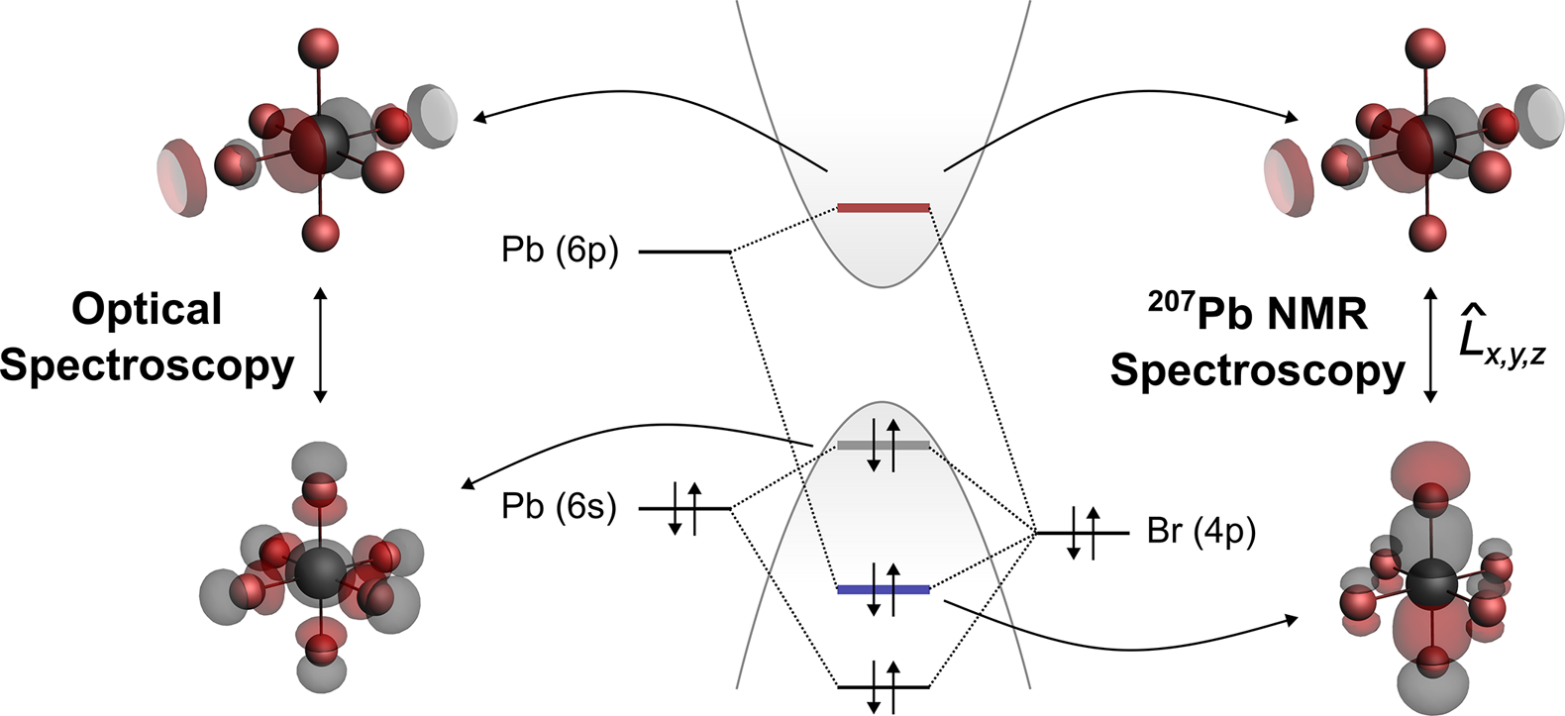
Simplified depiction of the electronic structure of APbBr_3_ showcasing the molecular orbitals involved in the optical band gap (left schematic) and the molecular orbitals responsible for the chemical shift as found by natural chemical shift analysis (right schematic).

Starting from the small cluster, i.e., PbBr_6_^4–^, the chemical shielding decomposes into σ_dia_ (∼9000 ppm) and σ_para+SO_ (∼−4000 ppm). The most significant contribution (∼−3000 ppm) to σ_para+SO_ arises from the coupling of a lower-lying molecular orbital (HOMO-n) of bonding character with the orthogonal LUMO, which is of antibonding nature (Figure 6). All other contributions to σ_para+SO_ are significantly smaller and are therefore not further considered in the discussion. Both molecular orbitals are 3-fold degenerate (along *x*, *y*, and *z*) and consist of a 6p orbital on Pb and a 4p orbital on each Br, either in antibonding or bonding configuration. Note that the HOMO does not have the appropriate symmetry to couple to the LUMO. Hence, the chemical shift is only indirectly connected to the optical band gap and is mainly driven by the energy of the LUMO (vide infra). To confirm the transferability of this finding to periodic structures, an analysis of the density of states (DOS) and crystal orbital overlap populations (COOP) is performed, visually placing the orbitals involved in σ_para_ at the same relative energy levels in both the cluster and periodic models and showing that the antibonding nature of both HOMO and LUMO is retained (Figure 5d,e and Figure S7). Apart from LHPs, similar correlations between the chemical shift and band gap have also previously been reported for conventional semiconductors such as Si, PbTe, and Hg_4–*n*_Cd_*n*_Te.^70−72^

**Figure 7:**
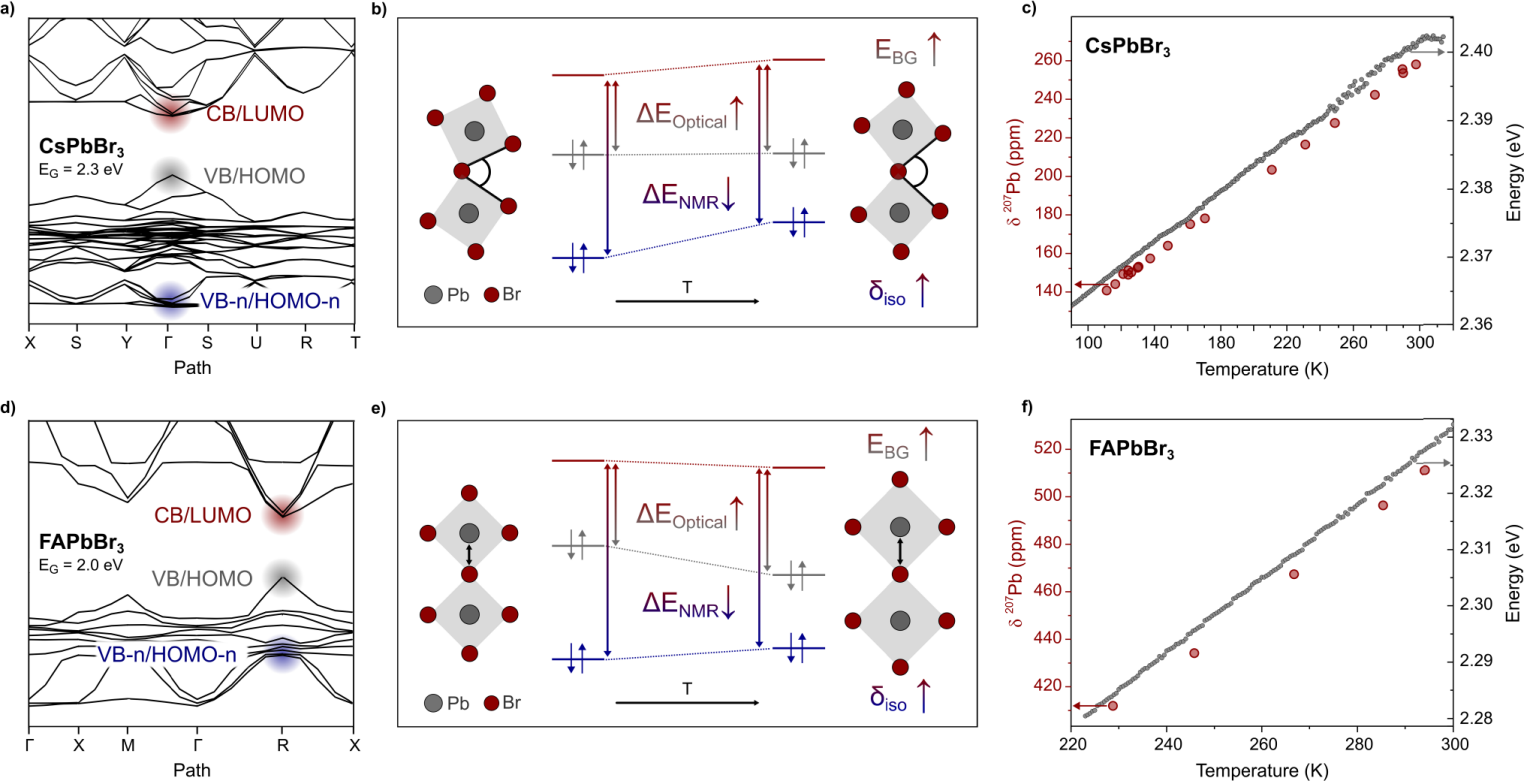
(a) Calculated electronic band structure of CsPbBr_3_ at room temperature, with the CB, VB and VB-n (red, gray and blue) highlighted by circular shaded areas. (b) Qualitative trend of the band energies relevant for the optical band gap (VB and CB) and chemical shift (VB-n and CB), obtained from the computational models, indicating a temperature-dependent increase in the optical band gap and decrease in the NMR “band gap”. (c) Temperature-dependent optical band gap (gray markers vs right axis) and ^207^Pb chemical shift (red markers vs left axis) for CsPbBr_3_. (d) The band structure diagram of FAPbBr_3_ shows reduced Δ*E*_Optical_ and Δ*E*_NMR_ compared to CsPbBr_3_. (e) Qualitative trend of the band energies upon thermal expansion of FAPbBr_3_. (f) Temperature-dependent optical band gap (gray markers; vs right axis) and ^207^Pb chemical shift (red markers; vs left axis) for FAPbBr_3_.

Having identified the relevant molecular orbitals contributing to the chemical shift, we set out to explain the difference in chemical shift between the room-temperature structures of CsPbBr_3_ and FAPbBr_3_, amounting to 259 ppm, as well as the increasing optical band gap and chemical shift with temperature. Starting from CsPbBr_3_, we identified the periodic analogs to the relevant molecular orbitals CB (LUMO), VB (HOMO), and VB-n (HOMO-n) in the band structure obtained from the optimized single crystal structure (Figure 7a). All analogous bands can be found at Γ, and the direct band gap of CsPbBr_3_ (2.3 eV) was found at the same point in reciprocal space. The energy of these bands is tracked to rationalize the trends of the spectroscopic descriptors across the temperature. To describe the thermal expansion in CsPbBr_3_, we calculate the chemical shielding using the large cluster to account for the altered tilting between neighboring octahedra. Upon increasing temperature, which manifests in reduced tilting between neighboring octahedra, the energy of both the CB and VB-n increases while the energy of the VB remains almost unchanged, resulting in an increase of the optical band gap (Figure 7b). These results can be reproduced by using both periodic and finite DFT with all three models (Figure S8). As the increase in energy of VB-n is steeper compared to the increase in CB, the difference in energy between both bands is reduced, resulting in an increase in the chemical shift (Figure 7b).

The chemical shift calculations also predict a linear increase in the chemical shift with temperature, which agrees with our experimental observations (Figure 7c and Figure S9). Note that including relativistic effects on the scalar and spin–orbit levels does not change the observed trend while significantly contributing to the overall shielding, i.e., σ_para_ and σ_SO._ We, therefore, included relativistic effects to the largest extent possible (spin–orbit for PbBr_6_ and scalar for Cs_56_Pb_27_Br_108_). The changes to the band energies are also consistent with the observed changes to the scalar coupling ^1^J_Pb–Br_: while the interaction between the 4p on Br and the 6p orbitals on Pb is increased upon heating, the interaction between the 4p orbitals on Br to the 6s orbital on Pb remains nearly unaffected resulting in a reduced s character of the bond between Pb and Br upon heating (Figure 7b). The change of the character of the bond is known to affect the strength of the scalar coupling through the Fermi contact term, which predicts an increasing coupling constant if the s character of the bond increases.^73^

The calculated band structure of FAPbBr_3_ shows a direct band gap of 2.0 eV at R (Figure 7d). The shift of the band gap is a result of crystal symmetry, while the smaller band gap for FAPbBr_3_ compared to CsPbBr_3_ was also observed experimentally. Notably, the energy difference between the CB and VB-n is reduced significantly compared to that of CsPbBr_3_. Considering that the chemical shift is inversely proportional to this energy difference, these computational results align with the experimental observation of a larger chemical shift in FAPbBr_3_ versus that in CsPbBr_3_ (513 and 254 ppm at room temperature). Based on this symmetry-driven shift of the energy levels, we expect that a first-order phase transition will result in a noticeable change in the chemical shift. This can be observed in MAPbBr_3_, which shows a sharp change of the chemical shift between 160 and 190 K in line with a first-order phase transition (Figure S5).

Tracking the energy levels of the bands in FAPbBr_3_ upon heating, which is mediated by an increase in the Pb–Br distance, we observe a lowering of the CB and VB energies, while the energy of the VB-n increases. The decrease of the VB energy is more pronounced than the decrease of the CB energy, leading to an overall increase of the optical band gap (Figure 7e). The difference in energy between the CB and the VB-n is reduced, resulting in an increasing chemical shift upon heating. While the origin of the effect is different, these changes to the band energies in FAPbBr_3_ also lead to both an increasing optical band gap and chemical shift (Figure 7f). This is further supported by chemical shift calculations, which show a linearly increasing chemical shift upon thermal expansion (Figure S10). The increased bond length in average cubic FAPbBr_3_ upon heating additionally rationalizes the greatly reduced ^1^J_Pb–Br_, as the bond is weakened by the increased bond distance, leading to reduced orbital overlap and associated reduced Fermi contact.

To summarize, the energy difference between the CB and VB probed by optical absorption spectroscopy increases in both cubic FAPbBr_3_ and orthorhombic CsPbBr_3_ upon thermal expansion. At the same time, the energy difference between the CB and VB-n, as probed by ^207^Pb NMR spectroscopy, decreases, leading to stronger paramagnetic deshielding and hence a larger chemical shift.

Comparing both cases, we want to highlight the implications of the energy shifts for optoelectronic devices that rely on a band alignment between the LHP and the charge-transport layers for efficient charge extraction.^74^ Tuning the relative band energies of the LHP with temperature can guide the selection of proper charge transport materials and may therefore increase the device performance. The observation of the correlation between the electronic structure and chemical shift underlines the unique power of NMR spectroscopy to elucidate and probe the local electronic structure,^52^ also for complex nanomaterials with small domain sizes that are inaccessible through XRD.

## Conclusions

With this study, we demonstrate the utility of variable-temperature ^207^Pb NMR spectroscopy to provide unique insight into the local electronic structure of LHPs when paired with DFT modeling. Through detailed analysis of temperature-dependent ^207^Pb chemical shifts and their correlation with the respective optical band gaps, we identified the molecular orbitals (CB and VB-n) that govern the chemical shift of Pb, turning the chemical shift into a spectroscopic descriptor. Tracking the energies of these bands across temperatures using DFT calculations based on single-crystal X-ray structures, we link the geometrical changes upon thermal expansion to our spectroscopic descriptors, turning the latter into precise lattice rulers, thanks to the intrinsically strong temperature dependencies of the optical absorption, NMR chemical shifts, and lattice parameters. We identify two thermal expansion mechanisms resulting in both an increasing band gap and chemical shift: Reduced tilting between neighboring corner-sharing PbBr_6_ octahedra in orthorhombic CsPbBr_3_ and elongation of the Pb–Br bonds in the average cubic FAPbBr_3_. The latter mechanism results in a 3-fold stronger thermal sensitivity of both the chemical shift and band gap than CsPbBr_3_. Using the local probing nature of NMR as a “lattice ruler”, we were also able to confirm both the mechanisms and the related changes to the electronic structures for colloidal QDs, circumventing the difficulties associated with diffraction-based analysis. Future work will focus on the broader mapping of thus-educated NMR descriptors across the defined libraries of QD shapes, compositions, and surface chemistries, with the goal of correlating with the respective optical properties.

## Experimental Section

### Preparation of Bulk Materials

#### CsPbBr_3_

Stochiometric amounts of CsBr and PbBr_2_ were loaded into a quartz ampule with an inner diameter of 10 mm. The ampule was evacuated and flame-sealed before being placed into a muffle furnace at 700 °C overnight. The resulting orange polycrystalline material was loaded into a furnace with three separately heated zones. Initially, the ampule was moved to a hot zone (>600 °C) until the material was fully molten before slowly (1 mm/h) moving it out of the furnace. The resulting ingot was optically transparent with a light orange color; under polarized light, some thermal cracking and twinning could be observed.

#### FAPbBr_3_

FAPbBr_3_ crystals were grown using inverse-temperature crystallization. 0.825 g of FABr and 2.57 g of PbBr_2_ were dissolved in a mixture of 5 mL of DMF and 5 mL of GBL at room temperature. The solution was then filtered through a 22 μm-PTFE filter and heated in an oil bath until the solution reached 60 °C. The temperature was then slowly increased to 70 °C until small orange crystals formed. The solution was kept at 70 °C for about 2 h or until the crystals stopped growing. The millimeter-sized crystals were then extracted from the solution and washed three times with toluene.

### Preparation of Quantum Dots

The synthesis procedure of the QDs and ligands was adapted from the literature.^20^ Initially, the PbBr_2_-TOPO precursor was diluted with *n*-hexane and stirred in an open flat-bottom flask. To this solution, either Cs-DOPA or FA-DOPA-OA precursor was swiftly injected. After the particle growth stabilized, a phosphoethanolamine-derived ligand was added to stabilize the QDs. The QDs were purified by the addition of an antisolvent (ethyl acetate:acetonitrile, 2:1 v:v), centrifugation, and redispersion of the precipitate in *n*-hexane. The purification was repeated two times in total. The size of the resulting QDs was controlled through the concentration of the precursors.

## Supporting Information Available

Additional preparation and measurement details, supplementary figures, and computational details (PDF)
