# Supplementary material for: Temperature-Dependent 207Pb Nuclear Magnetic Resonance Spectroscopy: A Spectroscopic Probe for the Local Electronic Structure of Lead Halide Perovskites
Source: Chem Mater. 2025 May 2;37(9):3443–54. doi: 10.1021/acs.chemmater.5c00354 (PMC12079797; doi:10.1021/acs.chemmater.5c00354)
Supplement: Supplementary file 1 — cm5c00354_si_001.pdf [file cm5c00354_si_001.pdf]

Supporting Information for:

Temperature-Dependent ^{207}Pb Nuclear Magnetic Resonance Spectroscopy: A Spectroscopic Probe for the Local Electronic Structure of Lead Halide Perovskites

Sebastian Sabisch[1,2], Marcel Aebli[1,2], Andrii Kanak[1,2], Viktoriia Morad[1,2], Simon C. Boehme[1,2], Michael Wörle[1], Leon G. Feld[1,2], Christophe Copéret[1] and Maksym V. Kovalenko[1,2]*

[1] ETH Zürich, Department of Chemistry and Applied Biosciences, Vladimir-Prelog-Weg 1-5, Zürich CH-8093, Switzerland

[2] Empa-Swiss Federal Laboratories for Materials Science and Technology, Überlandstrasse 129, Dübendorf CH-8600, Switzerland

Email: mvkovalenko@ethz.ch

METHODS

Single crystal growth

$CsPbBr_3$. Stochiometric amounts of CsBr (99.999%, Sigma-Aldrich) and $PbBr_2$ (prepared in-house) were loaded into a quartz ampule with an inner diameter of 10 mm. The ampule was evacuated and flame-sealed before being placed into a muffle furnace at 700 °C overnight. The resulting orange polycrystalline material was loaded into a Bridgman furnace with three separate heated zones. Initially, the ampule was moved to a hot zone (> 600 °C) until the material was fully molten before slowly (1 mm/h) moving it out of the furnace. The resulting ingot was optically transparent with a light orange colour, under polarized light some thermal cracking and twinning could be observed.

$FAPbBr_3$. $FAPbBr_3$ crystals were grown using inverse-temperature crystallization.[1] 0.825 g of FABr (> 99.99 %, GreatCell Solar Materials) and 2.57 g $PbBr_2$ (99 %, Sigma-Aldrich) were dissolved in a mixture of 5 mL DMF (Sigma-Aldrich > 99 %) and 5 mL GBL (99+ %, Arcos Organics) and stirred at room temperature until dissolved. The solution was then filtered through a 22µm-PTFE filter and heated in an oil bath until the solution reached 60 °C. The temperature was then slowly increased to 70°C until small crystals formed. The solution was kept at 70°C for about 2 hours or until the crystals stopped growing. The millimeter-sized crystals were then extracted from the solution and washed three times with toluene.

Fabrication of thin films

$CsPbBr_3$. A Bridgman-grown single crystal of $CsPbBr_3$ was gently ground into a fine powder in a nitrogen-filled glove box. The deposition material was placed in a crucible in a Radak source. Quartz glass was used as a substrate to enable proper heat conductivity. The substrate was ultrasonically cleaned in Hellmanex® III (2% in water), deionized water, acetone, and isopropanol for 15 min at each stage, followed by UV ozone treatment for 10 min. The thickness of the fabricated film was controlled by the mass of the deposition material (200 mg of starting material to obtain 100 nm perovskite film). The vacuum of the evaporation chamber was reduced to 10^{-6} Torr. The substrate temperature was controlled to be 20 °C. The deposition temperature was in the range 400-500 °C. The deposition rate was 0.6 Å/s. The substrate rotation velocity was 10 rpm. After the evaporation, the film was annealed at room temperature in a nitrogen-filled glove box for 1 week.

$FAPbBr_3$. FABr (> 99.99 %, GreatCell Solar) and $PbBr_2$ (99 %, Sigma-Aldrich) were dissolved in a mixture of DMF (Sigma-Aldrich > 99 %) and DMSO (Sigma-Aldrich > 99.5 %) (90:10) in a stoichiometric ratio to yield a 1.4 M solution. Both solvents were dried over 4 Å molecular sieves prior to use. The quartz substrate was cleaned by sonication in deionized water, acetone, and isopropanol for 10 minutes. The substrate was subsequently exposed to ozone plasma for 10 minutes before introduction into a nitrogen-filled glovebox. The ink was spin coated onto the substrate at 4000 rpm and rotated for another 60 seconds before annealing the substrate on a hotplate at 80°C for 10 minutes.

Synthesis of QDs

Chemicals. Lead(II) bromide ($PbBr_2$; 99.998%, Alfa), trioctylphosphine oxide (TOPO; 90%, Strem), formamidine acetate (FAOAc; 99% abcr), cesium carbonate (Cs_2CO_3),

diisooctylphosphinic acid (DOPA; 90%, Sigma-Aldrich), oleic acid (OA; 90%, Sigma-Aldrich), n-octane (>99%, for synthesis, Roth), n-hexane (>97%, HPLC, Sigma-Aldrich), toluene, mesitylene, acetonitrile, ethylacetate. The 2-octyl-1-dodecyl phosphoethanolamine (C8C12 PEA) was used as a capping ligand for colloidal QDs and synthesized as described in previous literature.[2] All the chemicals were used as received without further purification. Solvents were dried with molecular sieves (2 Å) prior to use.

Stock solutions of precursors. $PbBr_2$-TOPO precursor was prepared by mixing $PbBr_2$ (0.2 mmol) and TOPO (1 mmol) in n-octane (2.5 ml) at 120°C on a hotplate in the air until everything dissolves. After cooling to room temperature, the solution was diluted with n-hexane to reach a 0.067 M concentration. FA-DOPA-OA precursor 0.06 M: FAOAc (0.6 mmol), DOPA (3 ml), and OA (2 ml) were added to 5 ml n-octane and heated to 120°C in the air until the salt dissolved. Cs-DOPA precursor, 0.02 M: Cs_2CO_3 (0.3 mmol) and DOPA (1 ml) mixed in n-octane (2 ml) and heated to 120°C in the air until the salt dissolves and gas evolution stops. After cooling to room temperature, the solution was diluted with n-hexane (27 ml) to reach a 0.02 M concentration.

$CsPbBr_3$ NCs. $PbBr_2$-TOPO precursor (1300 uL, 0.067 M) was diluted with n-hexane (5 ml) and stirred in an open flat-bottom flask on a stirring plate. To this solution, Cs-DOPA precursor (1500 uL, 0.02 M) was swiftly injected. After 60 seconds, C8C12-PEA ligand (10 mg in 100 uL of mesitylene) was added. QDs were purified by addition of 2 eq. of antisolvent (ethylacetate:acetonitrile, 2:1 v:v), centrifugation (at maximum centrifugation speed for 30 s) and redispersion of the precipitate in n-hexane. The purification was repeated two times in total.

$FAPbBr_3$ NCs. $PbBr_2$-TOPO precursor (1950 uL, 0.067 M) was diluted with n-hexane (30 ml) stirred in an open flat-bottom flask on a stirring plate. To this solution, FA-DOPA-OA precursor (750 uL, 0.06 M) was swiftly injected. After 15s, C8C12-PEA ligand[1] (20 mg in 200 uL of

mesitylene) was added. The resulting colloid was concentrated on the rotary evaporator to 10 ml, and QDs were purified by addition of 1 eq. of antisolvent (ethylacetate:acetonitrile, 2:1 v:v), centrifugation (at maximum centrifugation speed for 30 s) and redispersion of the precipitate in n-hexane. The purification was repeated two times in total.

NMR details.

^{207}Pb NMR experiments were conducted on a 14.1 T wide-bore magnet equipped with a 3.2 mm double resonance low temperature MAS probe, an Avance III HD console and a low temperature MAS cabinet. Samples were prepare by gently crushing about 50 mg of larger crystals and mixing them with 1-2 mg of $Pb(NO_3)_2$ before packing them into a 3.2 mm zirconia or sapphire rotor. Spectra were acquired under magic angle spinning of 8 kHz using either one pulse excitation for sensitive bulk samples or an echo sequence with an echo delay of one rotation and an RF field strength of 55 kHz (60 W). The sample temperature was adjusted with the temperatures of the variable temperature, bearing and drive gas flows and confirmed using the chemical shift of $Pb(NO_3)_2$ after and equilibration period of 30 minutes. The initial room temperature measurement was used to reference the chemical shift to $Pb(NO_3)_2$. Recycle delay times were set to 1.5 times T_1 as determined by saturation recovery. Typical relaxation times were measured between 0.3 s and 1 s. All spectra were fitted using DmFit either using the implemented Gaussian/Lorentzian or the Jmultiplet models.

DFT details.

All computational models were initially based on the obtained experimental crystal structures. To preserve the effect of the temperature while still having the models under geometry optimization the optimization was carried out with periodic boundary conditions while restraining experimental

the lattice constants. The geometry optimizations were conducted using Slater-type orbitals with a basis set containing two zeta and one polarization function (DZP), the exchange functional proposed by Perdew, Burke and Ernzerhof[3] was used including the dispersion correction proposed by Grimme[4] as implemented in the AMS 2023 (BAND 2023.1) software suite. These calculations also include scalar relativistic effects using the zeroth order relativistic approximation. The optimized structures were used to obtain the band structures to correlate the relative band energies to their temperature.

Charged clusters containing 27 $PbBr_6$ ($A_{56}Pb_{27}Br_{108}^{2+}$ with A = Cs or FA) octahedra were cut from a supercell of the periodic model to conduct calculations with finite boundary conditions (using ADF2023). The NMR parameters were calculated for the central Pb atom, using the PBE functional, a DZP basis set, scalar relativistic approximations and a high numerical quality using the NMR package included in ADF 2023. Due to the large number of heavy atoms and the associated number of electrons a higher level of theory could not be achieved.

Natural chemical shift analysis was conducted on a smaller $PbBr_6$ cluster. These smaller clusters were run with a TZ2P basis set, including relativistic effects on the spin-orbit coupling level. The naturally bonding orbitals and naturally located molecular orbitals used for the NCS analysis were obtained using the ADFNBO 2022 code implemented in ADF 2022. We would like to acknowledge the Euler Cluster for providing the necessary computational resources.

Supporting Table 1. Results of the chemical shift calculation on $PbBr_6$

Pb-Br (Å)	σ_{dia} (ppm)	σ_{para} (ppm)	σ_{SO} (ppm)
3	10296.36	-3958.191	2846.327

3.05	10297.016	-3980.492	2803.304
3.1	10297.541	-4019.641	2753.777

Optical absorption.

Temperature-dependent optical-absorption spectra of $CsPbBr_3$ and $FAPbBr_3$ thin films on quartz substrates were acquired in transmission mode using a homebuilt fiber-based setup. The samples were mounted in a liquid-helium closed-cycle cold-finger cryostat (ARS; DE204AE; sample in vacuum) and probed via a broadband light source (OceanInsight; DH-2000-BAL), delivered to the sample free-space after passing through an optical fiber (Thorlabs; M112L02) and a collimator (Thorlabs; CVH100-COL and LA4647). The light transmitted through the sample was collected via a collimator (Thorlabs; CVH100-COL and LA4647) and a fiber bundle (Thorlabs; BFL200HS02) and subsequently analyzed via a broadband spectrometer (OceanInsight; HDX-UV-VIS). Absorption spectra were calculated from a reference measurement using the quartz substrate and corrected for dark counts. To record temperature-dependent spectra, the temperature was increased from 15 K to 300 K at a rate of 3 K/min while recording a spectrum every 20 s.

Single Crystal X-ray diffraction.

X-ray diffraction patterns were acquired using a Rigaku XtaLAB Synergy-S equipped with a Cu and Mo source, a HyPix-6000HE detector, and a cryostat. After the selection of suitable crystallites under polarized light, a small piece (30 x 40 x 90 um for $FAPbBr_3$ and 53 x 95 x 170 um for CsPbBr3) was cut off using a scalpel. The crystal was then mounted on a Kapton loop using high vacuum grease. The single-crystal experiments used Mo radiation for $FAPbBr_3$ and Cu radiation for $CsPbBr_3$. Structure solutions were performed using ShelXT,[5] refinements were carried out

using ShelXL.[6] At room temperature, a cubic unit cell of $FAPbBr_3$ in the space group *Pm*-3*m* was found, with a lattice constant of 5.9909(3) Å. The orthorhombic unit cell of $CsPbBr_3$ in the space group *Pmna* was found at room temperature to have the following unit cell parameters: a = 8.2538(3) Å, b = 11.7577(4) Å and c = 8.2109(3) Å. Single crystal diffraction experiments were performed between 100 K and 300 K with an interval of 20 K, resulting in a series of temperatures for $CsPbBr_3$ of 100 113, 133, 153, 173, 193, 213, 233, 253, 273 and 298 K. The temperature scan for $FAPbBr_3$ was carried out using temperatures of 100, 180, 200, 220, 240, 260, 280 and 298 K. The report for both room temperature structures can be found below, while all CIF files can be found in the supporting information.

Sample / Empirical Formula	$CN_2H_5PbBr_3$	$CsPbBr_3$
Formula weight	491.98	579.825
Temperature	292.99(10) K	292.94(10) K
Crystal system	cubic	orthorhombic
Space group	*Pm*-3*m*	*Pnma*
a	5.9909(3) Å	8.2538(3) Å
b	= a	11.7577(4) Å
c	= a	8.2109(3) Å
α = β = γ	90 °	90 °
Volume	215.02(3) $Å^3$	796.83(5) $Å^3$
ρ	3.815 g/cm^3	4.833 g/cm^3
μ	33.460 mm^{-1}	92.968 mm^{-1}
F(000)	211.0	968.0
Crystal size	0.09 x 0.04 x 0.03 mm^3	0.05 x 0.1 x 0.17 mm^3
Radiation	Mo Kα (λ = 0.71073 Å)	Cu Kα (λ = 1.54184 Å)
2Θ range	6.802° to 70.214°	13.152° to 159.694°
Index ranges	-8 < h < 9, -9 < k 9, -8 < l < 9	-10 < h < 10, -15 < k 14, -10 < l < 10
Reflections collected	1917	8125
R_{int}	0.1078	0.0864
R_{sigma}	0.0337	0.0449
Restraints	0	0
GoF on F^2	1.201	1.117

R indices ($I > 2\sigma(I)$)	$R_1 = 0.0480$, $wR_2 = 0.0779$	$R_1 = 0.0705$, $wR_2 = 0.2134$
R indices (all data)	$R_1 = 0.0598$, $wR_2 = 0.0797$	$R_1 = 0.0740$, $wR_2 = 0.2181$
Largest diff. peak/hole	2.09/-2.27 eÅ^{-3}	4.99/-4.69 eÅ^{-3}

Fractional Atomic coordinates (x10^4) and equivalent isotropic displacement parameters (Å^{2}x10^3):

$CN_2H_5PbBr_3$	x	y	z	U(eq)
Pb	0	0	0	40.3(5)
Br	5000	0	0000	87.3(12)
C	4150 (110)	5300 (400)	4900 (400)	20 (20)
N1	5300(400)	5400 (200)	3010 (140)	20 (20)
N2	4560 (160)	3960 (180)	6540 (140)	20 (20)
$CsPbBr_3$				
Pb	0	5000	5000	17.4(5)
Cs	5308(5)	2500	5071(3)	70.5(9)
Br1	2933(3)	4756(2)	7056(3)	57.5(9)
Br2	36(5)	7500	5473(8)	64.4(13)

Anisotropic displacement parameters (Å^{2}x10^3):

$CN_2H_5PbBr_3$	U_{11}	U_{22}	U_{33}	U_{23}	U_{13}	U_{12}
Pb	40.3(5)	40.3(5)	40.3(5)	0	0	0
Br	112.5(19)	112.5(19)	112.5(19)	0	0	0
$CsPbBr_3$						
Pb	23.7(7)	7.2(7)	21.7(7)	0.5(18)	0.1(2)	1.1(2)
Cs	90.3(17)	40.9(14)	80(2)	0	33.5(12)	0
Br1	50.6(13)	74.3(16)	47.7(12)	-6.6(10)	-27.7(10)	8.6(11)
Br2	101(3)	7.0(13)	97(3)	0	13(2)	0

Note to the structure solution of $FAPbBr_3$: Due to the disorder on the organic cation the CN_2H_5 fragment was approximated by a rigid fragment.

Powder X-ray diffraction.

Powder diffraction patterns were obtained using a STADI-P (STOE) diffractometer in a Debeye-Scherrer geometry. The diffractometer was equipped with a $AgK_{\alpha 1}$ ($\lambda = 0.55941$ Å) source and 4

Mythen2 1k Si-strip detectors (DECTRIS). The samples were prepared by grinding the crystals into a fine powder and filling the powder into a 0.3 mm glass capillary. A range between 0° - 138° was acquired over a period of 56 h. Prior to processing the edges of the diffractogram influenced by the beam stop were removed.

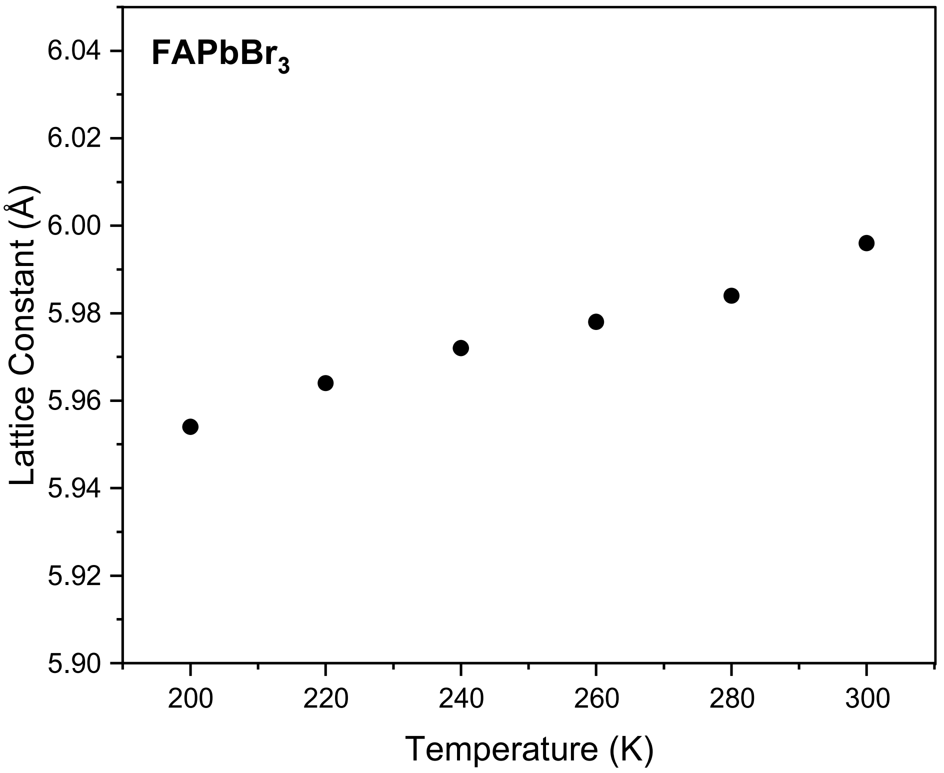

Figure S1. Lattice constant of $FAPbBr_3$ obtained from the single-crystal X-ray diffraction experiments showing a monotonous increase with temperature. As all obtained structures were described in a cubic crystal system, the expansion is, per definition, isotropic.

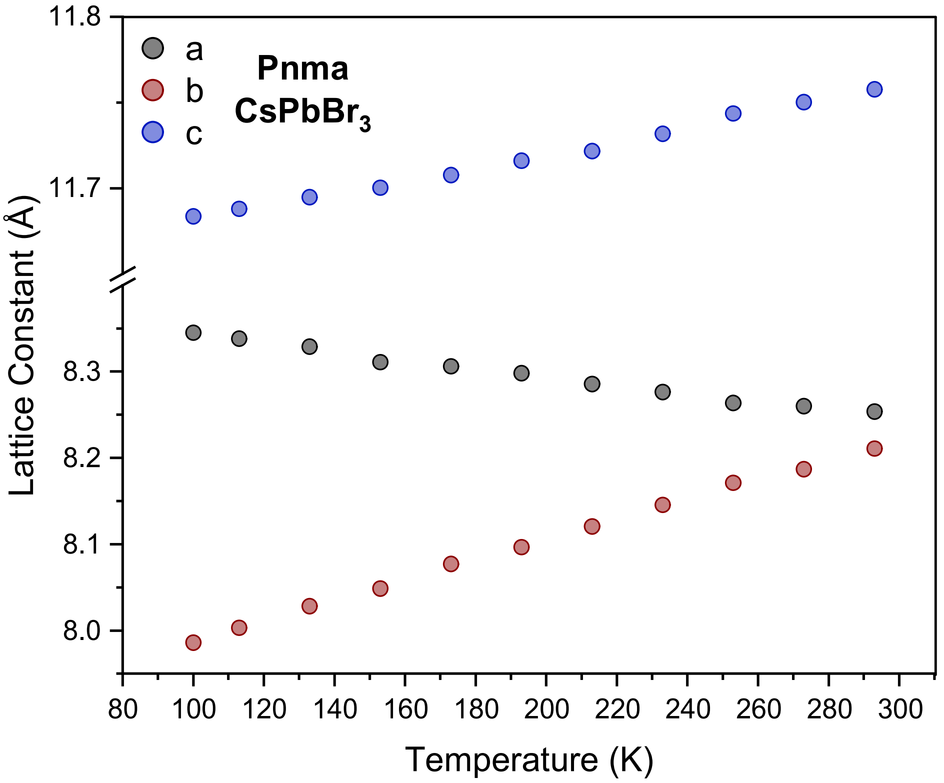

Figure S2. Lattice constants of $CsPbBr_3$ obtained from the single crystal X-ray diffraction experiments. All lattice constants show a monotonous behaviour, both the b and c axes elongate with temperature while the a axis contracts. Overall, these changes to the lattice constants still lead to an anisotropic expansion of the unit cell with temperature.

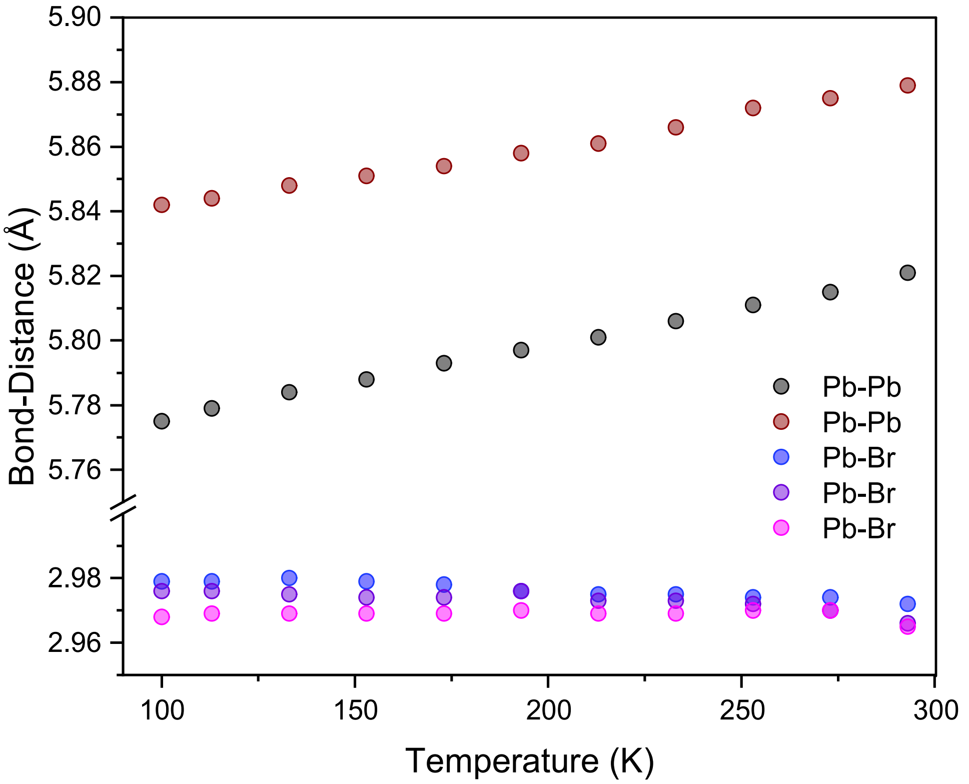

Figure S3. Interatomic distances in $CsPbBr_3$ obtained from the single crystal X-ray diffraction experiments. There are two distinct distances between pairs of Pb atoms which correspond to the equatorial and axial directions, this is also reflected in the difference in the Pb-Br-Pb angles along the same directions (vide supra). While the Pb-Pb distances increase with temperature the Pb-Br distances remain nearly unchanged regardless of their orientation in the unit cell.

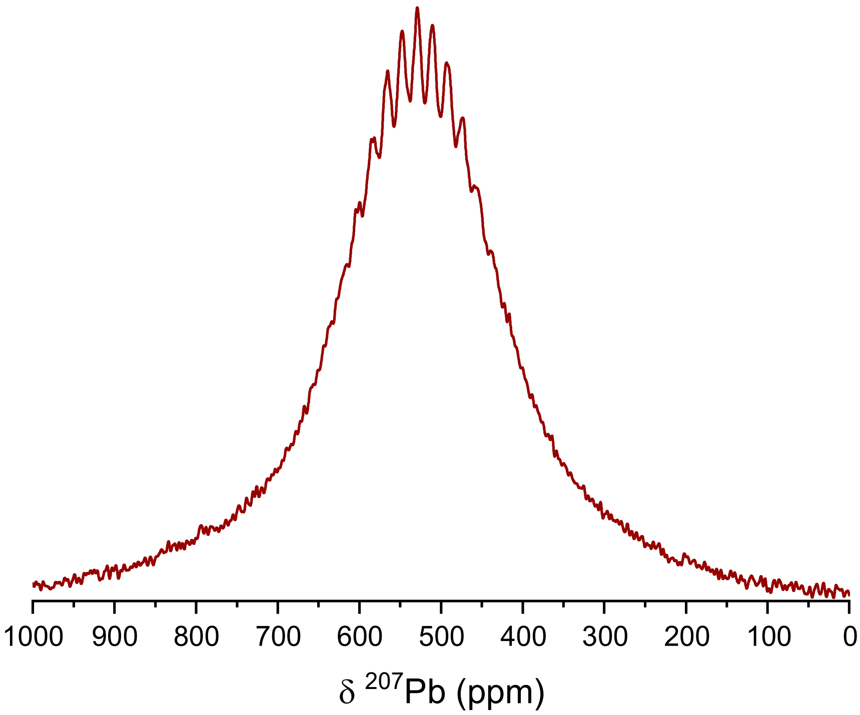

Figure S4. A ^{207}Pb MAS NMR spectrum of $FAPbBr_3$ obtained using a room temperature 3.2 mm double resonance probe and an Avance III HD spectrometer at 14.1 T. The spinning rate was set to 8 kHz and a total of 128k scans were acquired with a recycle delay of 1s. The spectrum shows a single line at 530 ppm with a pronounced j-coupling with a coupling constant of 2265 Hz.

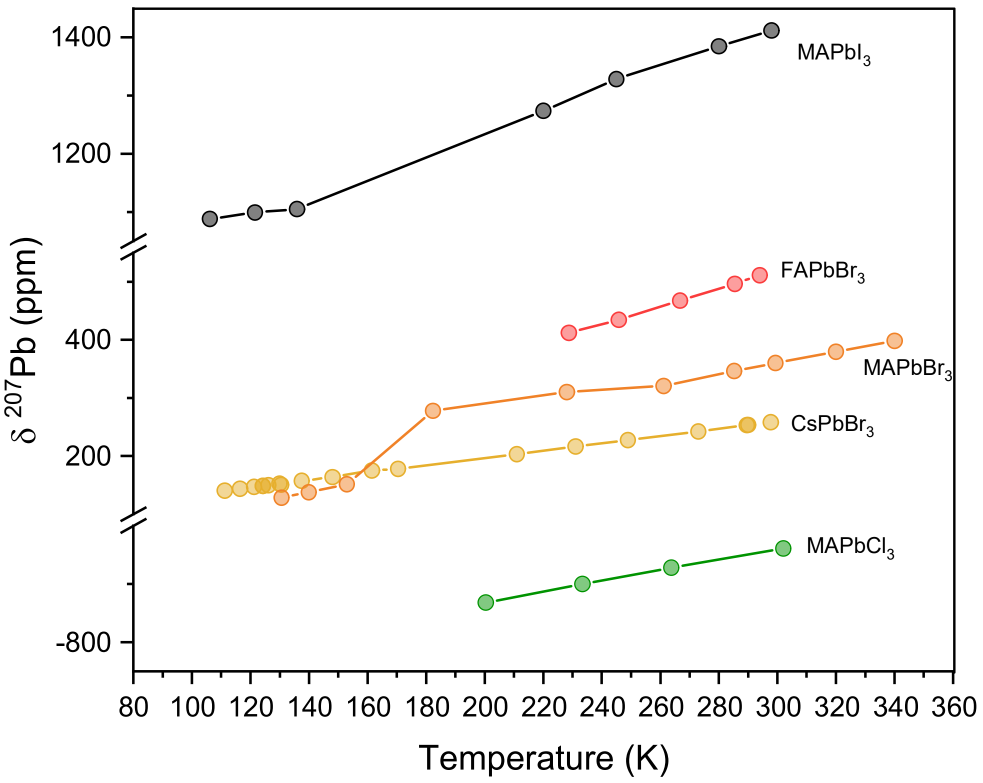

Figure S5. ^{207}Pb chemical shift of several LHPs across temperature. All spectra were obtained at a spinning frequency of 8 kHz using a double resonance 3.2 mm low temperature probe. The spectra of $MAPbBr_3$ were obtained at a field of 11.7 T, all other spectra were obtained at 14.1 T. The chemical shift for all compositions increases with temperature. For some compositions phase transitions can be observed as discontinuities in the trends ($MAPbBr_3$ at 160 K).

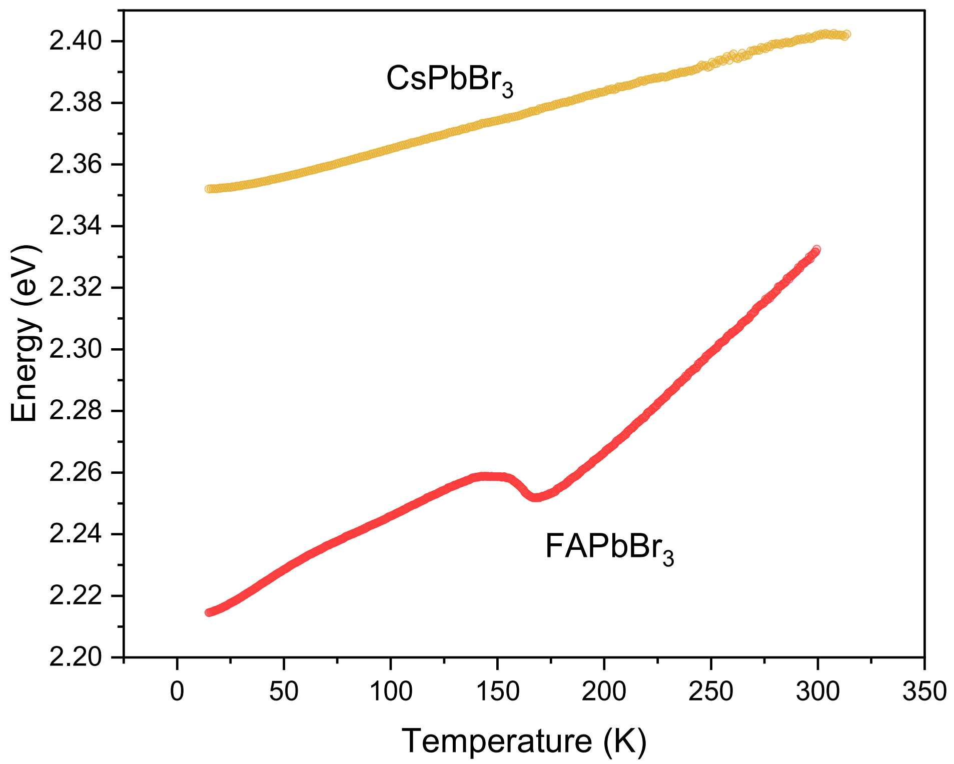

Figure S6. Energy of the excitonic feature of thin-films of $CsPbBr_3$ and $FAPbBr_3$ between 15K and room temperature as obtained from optical absorption spectroscopy.

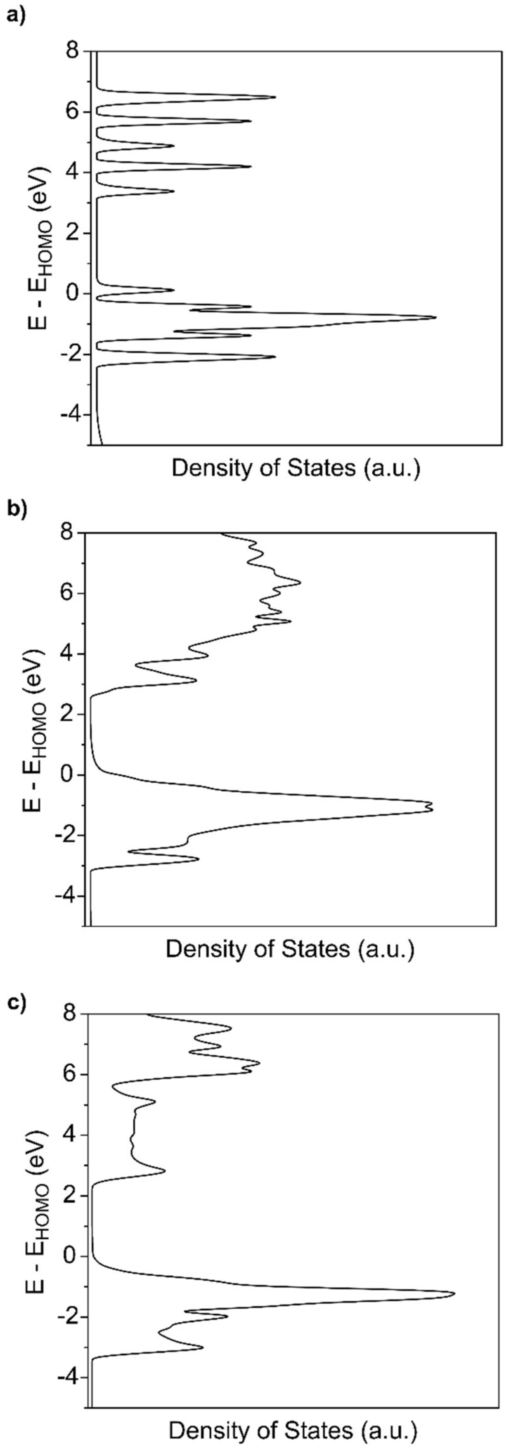

Figure S7. Density of states obtained of the small cluster model (a), large cluster model (b) and periodic structure (c) obtained from DFT calculations under periodic conditions.

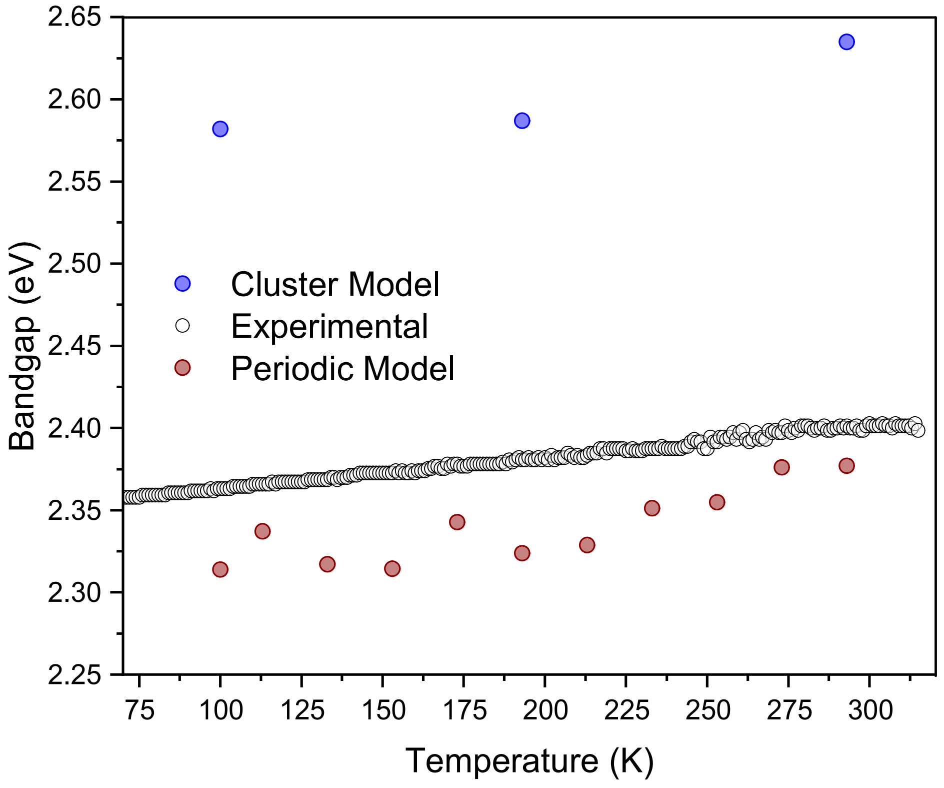

Figure S8. Calculated and experimental bandgaps of $CsPbBr_3$ between 100 K and 300 K. All traces show an increase of a similar slope with increasing temperature. The calculated bandgap of the cluster model is larger than the periodic model due to confinement effects. This offset is expected to decrease with increasing cluster size.

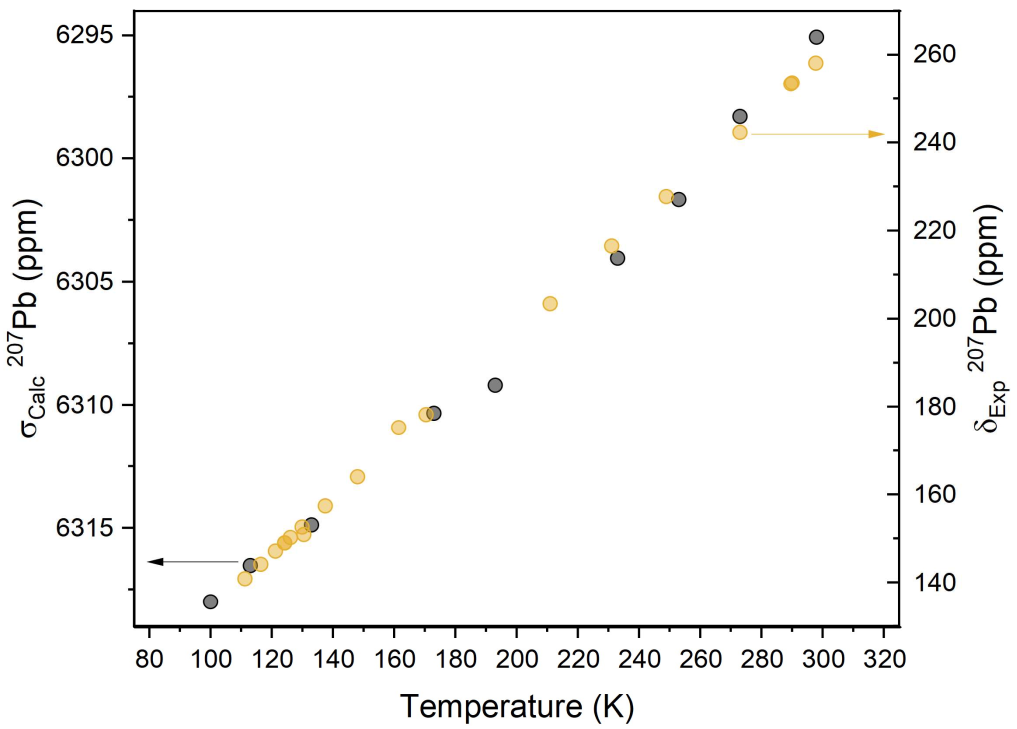

Figure S9. Experimentally obtained chemical shift and calculated chemical shielding of CsPbBr3 between 100 K and 300 K. The calculations were carried out using the larger cluster model based on the periodically optimized single crystal structures. Both the experimental and calculated values show a monotonous behaviour against temperature. Similar to the smaller cluster model the calculation underestimates the change in the chemical shielding due to limitations to the level of theory used in the calculations (vide supra).

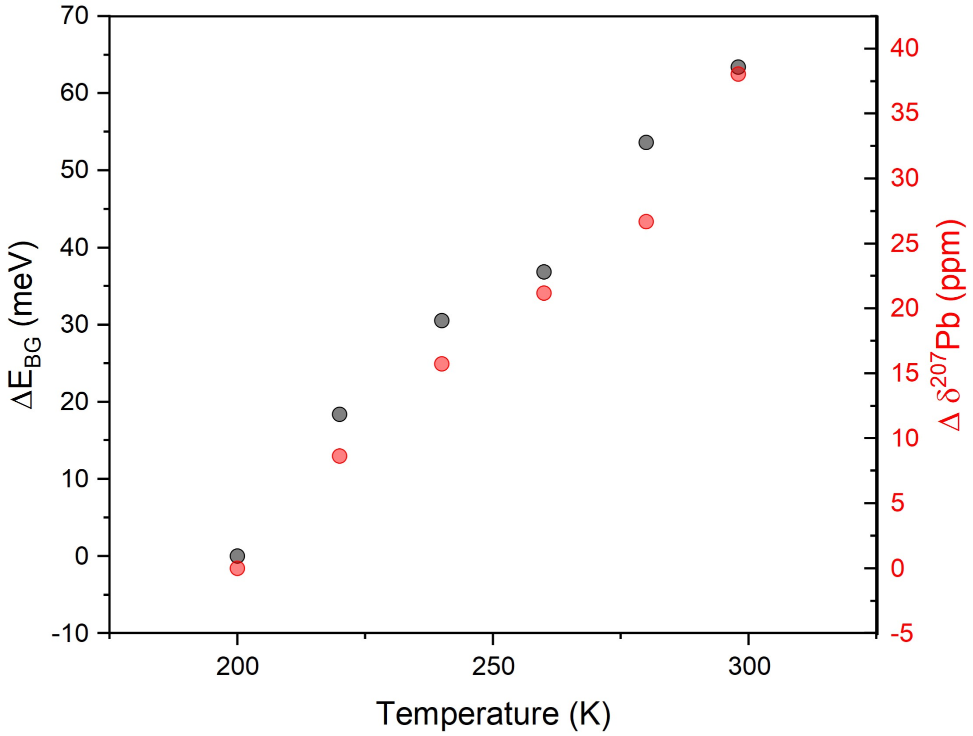

Figure S10. Calculated bandgap and chemical shifts of $PbBr_6^{4-}$. The Pb-Br distances were obtained from the structure of $FAPbBr_3$ at different temperatures. The differences are plotted relative to the values obtained from the 200 K structure. Both the bandgap and chemical shift increase with the temperature. While the differences in the bandgap come close to the experimental values the change in the chemical shift is underestimated due to the limitations of the model.

REFERENCES

(1) Saidaminov, M. I.; Abdelhady, A. L.; Murali, B.; Alarousu, E.; Burlakov, V. M.; Peng, W.; Dursun, I.; Wang, L.; He, Y.; Maculan, G.; Goriely, A.; Wu, T.; Mohammed, O. F.; Bakr, O. M. High-Quality Bulk Hybrid Perovskite Single Crystals within Minutes by Inverse Temperature Crystallization. *Nature Comm.*, **2015**, *6* (1). DOI: 10.1038/ncomms8586

(2) Morad, V.; Stelmakh, A.; Svyrydenko, M.; Feld, L. G.; Boehme, S. C.; Aebli, M.; Affolter, J.; Kaul, C. J.; Schrenker, N. J.; Bals, S.; et al. Designer Phospholipid Capping Ligands for Soft Metal Halide Nanocrystals. *Nature* **2024**, *626* (7999), 542-548. DOI: 10.1038/s41586-023-06932-6

(3) Perdew, J. P.; Burke, K.; Ernzerhof, M. Generalized Gradient Approximation Made Simple. *Phys. Rev. Lett.* **1996**, *77* (18), 3865-3868. DOI: 10.1103/PhysRevLett.77.3865

(4) Grimme, S.; Ehrlich, S.; Goerigk, L. Effect of the damping function in dispersion corrected density functional theory. *J. Comput. Chem.* **2011**, *32* (7), 1456-1465. DOI: 10.1002/jcc.21759

(5) Sheldrick, G. SHELXT - Integrated space-group and crystal-structure determination. *Acta Crystallogr. Sect. A* **2015**, *71* (1), 3-8. DOI: 10.1107/S2053273314026370

(6) Sheldrick, G. Crystal structure refinement with SHELXL. *Acta Crystallogr. Sect. C* **2015**, *71* (1), 3-8. DOI: 10.1107/S2053229614024218
